# Cross-dataset transcriptomics–proteomics analysis identifies *Tlr2*, *Anxa5*, and *Grin2a* as candidate mouse-brain signatures associated with chronic cerebral hypoperfusion

**DOI:** 10.3389/fneur.2026.1911864

**Published:** 2026-10-01

**Authors:** Xiang Fan, Meng-Yu Zhong, Huan Liu, Hao Tao, Yi Li, Xun Wu, Ying-jie Li, Bing-Hu Li, Neng-Wei Yu

**Affiliations:** 1 School of Medicine, University of Electronic Science and Technology of China, Chengdu, China; 2 Department of Neurology, Sichuan Provincial People's Hospital, Chengdu, China; 3 School of Clinical Medicine, Southwest Medical University, Luzhou, China

**Keywords:** BCAS, biomarkers, chronic cerebral hypoperfusion, proteomics, transcriptomics

## Abstract

**Background:**

Chronic cerebral hypoperfusion (CCH) is a neurological disorder characterized by persistent insufficiency of cerebral blood flow, yet specific biomarkers and mechanistic insights are lacking.

**Methods:**

This study integrated transcriptomic and proteomic multi-omics analyses, along with behavioral assessments and immunofluorescence validation in a bilateral common carotid artery stenosis (BCAS) mouse model, to identify and validate candidate multi-omics biomarkers of CCH and explore their potential druggability. Leveraging public transcriptomic datasets and in-house proteomic data, differential expression analysis, protein-protein interaction network construction, and five topological algorithms identified *Tlr2*, *Anxa5*, and *Grin2a* as candidate biomarkers.

**Results:**

BCAS mice exhibited spatial memory impairments in behavioral tests. Immunofluorescence revealed upregulated *TLR2* and *ANXA5*, downregulated *GRIN2A,* and increased TLR2-IBA1 co-localization indicating microglial activation. *Tlr2* and *Anxa5* positively correlated with infiltrating immune cells, whereas Grin2a showed a negative correlation. A putative ceRNA network and in silico molecular docking suggested strong binding of pirinixic acid to both TLR2 and ANXA5.

**Discussion:**

*Tlr2*, *Anxa5*, and *Grin2a* can serve as candidate multi-omics signature molecules of CCH in the mouse brain, with consistent alterations at the transcriptomic, proteomic, and tissue levels.

## 1 Introduction

Chronic cerebral hypoperfusion (CCH) refers to a persistent state of inadequate cerebral blood flow resulting from intracranial arterial stenosis or occlusion and cerebral small vessel disease, the crux of which lies in the multi-systemic, progressive pathological changes induced by long-term hypoperfusion (1). Epidemiological studies have revealed that approximately 30% of individuals aged 45–50 years have a history of CCH, with this proportion rising to as high as 70% among those over 80 years of age (2). CCH can induce or facilitate a spectrum of neurological disorders and dysfunctions, including vascular cognitive impairment (VCI), vascular dementia (VaD), Alzheimer’s disease (AD), white matter hyperintensities (WMH), white matter microstructural disruption, and demyelination (3–5). The pathogenic mechanisms underlying CCH are intricate, with multiple interwoven pathways—such as blood–brain barrier disruption, neuroinflammation, oxidative stress, and mitochondrial dysfunction—converging to exacerbate neurodegenerative progression. Moreover, the clinical manifestations of CCH are often insidious and thus susceptible to oversight (6, 7).

Although the clinical ramifications of CCH have been widely acknowledged, there remains a dearth of biomarkers with high specificity and sensitivity, which substantially hampers timely disease management and effective therapeutic intervention. Current diagnostic modalities rely predominantly on neuroimaging (e.g., MRI white matter hyperintensities, cerebral blood flow perfusion assessment) and neuropsychological testing, neither of which enables early intervention at the molecular level (8). Single-omics analyses are inherently limited by a unidimensional information framework, elevated false-positive rates, and incomplete mechanistic interpretation (9). For instance, thousands of differentially expressed genes identified from transcriptomic data alone may encompass substantial noise that fails to translate into functional proteins, leaving protein-level alterations unaccounted for. In contrast, multi-omics integration strategies can enhance the accuracy and reliability of biomarker discovery while providing more comprehensive and in-depth mechanistic insights (10).

In the present study, we aimed to systematically identify candidate biomarkers of CCH by integrating publicly available transcriptomic data with data-independent acquisition (DIA) proteomic data generated from a bilateral common carotid artery stenosis (BCAS) mouse model established by our group. Building upon bioinformatics screening, candidate biomarkers were further validated via immunofluorescence staining in mouse brain tissues. Furthermore, through multi-dimensional analyses such as gene set enrichment analysis (GSEA), immune infiltration profiling, ceRNA network construction, and molecular docking, we sought to elucidate the biological functions and drug-targeting potential of the candidate biomarkers, thereby providing novel research perspectives for the diagnosis and targeted therapy of CCH, which remain to be validated in future studies.

## 2 Materials and methods

### 2.1 Animals and BCAS model

Twelve male C57BL/6 J mice (10 weeks of age, 20–25 g) were obtained from Spbio (Beijing) Biotechnology Co., Ltd. All animal experiments were conducted in the animal facility of Sichuan Lilai Sinuo Biotechnology Co., Ltd. (license number: SYXK [Sichuan] 2024–0287). The mice were randomly assigned to either a sham-operated group (sham, *n* = 6) or a BCAS model group (*n* = 6). Multiple studies have shown that the BCAS mouse model reliably mimics CCH, and the BCAS procedure was performed as described by Kakae et al. (11): bilateral common carotid artery stenosis was induced using microcoils (internal diameter, 0.18 mm) made of copper wire. In the sham group, the carotid arteries were exposed via cervical incision and then sutured without microcoil placement. Postoperatively, all animals were housed in standard cages with ad libitum access to food and water under a 12-h light/dark cycle at 20–26 °C and 30–70% relative humidity. All animal procedures in this study were reviewed and approved by the Animal Ethics Committee of Sichuan Academy of Medical Sciences & Sichuan Provincial People’s Hospital (No. 2025479) and were performed in strict compliance with relevant animal welfare regulations.

### 2.2 Behavioral tests

The open field test (OFT) was performed 28 days post-surgery to evaluate locomotor activity and anxiety-like behavior (12). The test was conducted in a white open field arena (50 cm × 50 cm × 40 cm). Prior to testing, animals were allowed to habituate to the testing room for 30 min. Each mouse was placed in the arena and permitted to freely explore for 10 min, with locomotor trajectories automatically recorded and analyzed using the Noldus EthoVision XT video tracking system (Noldus Information Technology, Wageningen, The Netherlands). The primary outcome measures included total distance traveled (cm), mean velocity (cm/s), and the frequency of entries into and cumulative duration spent in the central zone. The arena floor was thoroughly cleaned with 75% ethanol between trials to eliminate residual olfactory cues.

Following the OFT, the Morris water maze (MWM) was conducted to assess spatial learning and memory (13). The apparatus consisted of a circular pool (120 cm in diameter, 50 cm in depth) filled with water maintained at 22 ± 1 °C and rendered opaque by the addition of white nontoxic pigment. During the acquisition phase (days 1–5), mice underwent daily hidden-platform training with four trials from different starting positions (N, E, SE, and NW), and escape latency was recorded for each trial. On day 6, the platform was removed for the probe trial, during which escape latency, number of platform crossings, and time spent in the target quadrant were recorded over a 60-s period (14).

### 2.3 Tissue collection and sample preparation

Following the completion of behavioral testing, mice were anesthetized by intraperitoneal injection of sodium pentobarbital (50 mg/kg). After the loss of righting reflex and confirmation of absent nociceptive responses, the animals were euthanized by cervical dislocation, and cessation of heartbeat and respiration was verified. Brain tissues from three randomly selected mice per group were immediately placed in cryovials, snap-frozen in liquid nitrogen, and transported on dry ice for subsequent proteomic analysis. The remaining brain tissues (three mice per group) were collected and embedded in paraffin for immunofluorescence staining.

### 2.4 Immunofluorescence staining

Immunofluorescence staining was performed on paraffin-embedded coronal brain sections at the cortical region. After deparaffinization in xylene and rehydration through a graded ethanol series, antigen retrieval was performed by microwave heating in citrate buffer (pH 6.0) for 20 min. Endogenous peroxidase activity was quenched with 3% H₂O₂ for 25 min at room temperature, and non-specific binding was blocked with 3% bovine serum albumin (BSA) for 30 min. For TLR2 and IBA1 double staining, a tyramide signal amplification (TSA)-based sequential multiplex approach was used. Sections were first incubated with rabbit anti-IBA1 antibody (Zen Bio, R382207, 1:200) overnight at 4 °C, followed by HRP-conjugated goat anti-rabbit IgG (Servicebio, GB23303, 1:100) for 50 min at room temperature. TSA-FITC (Servicebio, G1222, 1:500) was then applied for 10 min to visualize IBA1 in green. The first-round antibody complex was removed by microwave heating in citrate buffer (10 min heating, 8 min rest, 10 min heating). Sections were then incubated with rabbit anti-TLR2 antibody (Abcam, ab213676, 1:200) overnight at 4 °C, followed by Cy3-conjugated goat anti-rabbit IgG (Servicebio, GB21303, 1:200) for 30 min at 37 °C to visualize TLR2 in red. For ANXA5 and GRIN2A detection, sections were processed following a conventional immunofluorescence protocol. After blocking, sections were incubated overnight at 4 °C with rabbit anti-ANXA5 (Cusabio, CSB-PA06384A0Rb, 1:100) or rabbit anti-GRIN2A (Thermo Fisher Scientific, MA5-35255, 1:100), then with FITC-conjugated goat anti-rabbit IgG (Servicebio, GB22303, 1:100) for 30 min at room temperature. All sections were counterstained with DAPI for 10 min and mounted with anti-fade mounting medium. For each marker, two non-overlapping 200 × cortical fields per animal were captured using an Olympus VS200 slide scanner. The percentage of positive tissue area (% Positive Tissue) was quantified using the Halo image analysis platform (Indica Labs, USA). Positive signals appeared green (FITC) for ANXA5, GRIN2A, and IBA1, and red (Cy3) for TLR2, with nuclei in blue (DAPI). The values measured in these two fields were averaged to obtain a single value per animal, which served as the unit of statistical analysis (biological replicates: sham group, *n* = 3; BCAS group, *n* = 3). Raw per-animal values are provided in Supplementary Table S1.

### 2.5 Protein extraction and MS data acquisition

Mouse brain tissues were homogenized in pre-chilled lysis buffer and centrifuged at 14,000 × g to collect the supernatant. Protein concentration was determined using the bicinchoninic acid (BCA) assay, and sample quality was verified by SDS-PAGE. Proteins were reduced with TCEP, alkylated with iodoacetamide, and subsequently digested with mass spectrometry-grade trypsin at 37 °C overnight. The resulting peptides were desalted using StageTips and quantified with a NanoDrop spectrophotometer. Equal amounts of peptides from each sample were subjected to data-independent acquisition (DIA) analysis on a nano-liquid chromatography–tandem mass spectrometry system (Vanquish Neo coupled to Astral, Thermo Fisher Scientific, USA). Chromatographic separation was performed on an in-house packed C18 column (15 cm × 100 μm, 1.7 μm) with a gradient elution over 8 min, using mobile phase A (2% acetonitrile containing 0.1% formic acid) and mobile phase B (80% acetonitrile containing 0.1% formic acid). Mass spectrometry was operated in positive ion mode with a spray voltage of 2.2 kV. MS1 scans were acquired over an m/z range of 380–980, and MS2 scans over 150–2000 m/z. Raw data were processed using Spectronaut 18.0 (Biognosys, Switzerland) and searched against the mouse UniProt database^1^ with a false discovery rate (FDR) threshold of < 0.01. Protein relative quantification was performed based on peak area intensity.

### 2.6 Data collection

Transcriptomic datasets associated with CCH were retrieved from the Gene Expression Omnibus (GEO) database,^2^ including GSE210666 and GSE275195. The GSE210666 dataset, based on the GPL24247 platform, comprised transcriptomic data from 4 control and 4 CCH samples. The GSE275195 dataset, also based on the GPL24247 platform, included transcriptomic data from 3 control and 3 CCH samples. Proteomic data of brain tissues were derived from our in-house BCAS mouse model, consisting of 3 control and 3 model samples.

### 2.7 Identification of candidate genes

To assess the overall correlation between transcriptomic and proteomic samples and the clustering patterns within each group, pairwise correlation analysis and principal component analysis (PCA) were performed on both omics datasets. Differential expression analysis of the proteomic data was conducted using the limma package (v3.56.2) (15). Differentially expressed proteins (DEPs) were defined using the criteria of |log₂(fold change)| > 0.5 and *p* < 0.05, and the results were visualized as volcano plots and heatmaps. For the transcriptomic data, differential expression analysis between the CCH and control groups was performed separately for the GSE210666 and GSE275195 datasets using DESeq2 (v1.36.0) (16), with the same thresholds applied to identify differentially expressed genes (DEGs). Gene Ontology (GO) and Kyoto Encyclopedia of Genes and Genomes (KEGG) enrichment analyses of DEGs and DEPs were performed using the clusterProfiler package (v4.14.4) with a significance threshold of *p* < 0.05 (17). Finally, the upregulated and downregulated genes from the two transcriptomic datasets were intersected separately to identify candidate genes.

### 2.8 Identification of biomarkers

To further identify biomarkers, protein–protein interaction (PPI) analysis of the candidate genes was performed using the STRING database^3^ with a minimum confidence score of 0.4. Five topological algorithms within the cytoHubba plugin of Cytoscape—Stress, Degree, Maximal Neighborhood Component (MNC), Betweenness, and Edge Percolation Component (EPC)—were applied to identify hub genes in the PPI network (parameter: Top 10). The intersection of the results from all five algorithms was determined to define the final core gene set. The expression levels of the candidate genes were compared between the CCH and control groups using the Wilcoxon rank-sum test in the discovery transcriptomic dataset (GSE210666), the additional transcriptomic dataset (GSE275195), and the in-house proteomic dataset. Genes exhibiting significant differential expression (*p* < 0.05) with concordant expression trends across datasets were retained. Receiver operating characteristic (ROC) curves were then plotted using the pROC package in R, and the area under the curve (AUC) was calculated to quantitatively evaluate the diagnostic performance of each molecule in both transcriptomic datasets, with an AUC closer to 1 indicating superior discriminative capacity. Genes that were significantly differentially expressed at both the transcriptomic and proteomic levels and exhibited AUC values > 0.7 in these datasets were ultimately selected as candidate biomarkers for subsequent analyses (18).

### 2.9 Functional annotation of biomarkers

To identify potential KEGG pathways associated with the biomarkers, pre-ranked gene set enrichment analysis (GSEA) was performed based on the discovery dataset GSE210666. Gene sets from the GOBP and KEGG collections in the MSigDB database (*Mus musculus*) were utilized. For each biomarker, Pearson correlation coefficients with all other genes were computed, and genes were ranked in descending order of the correlation coefficients. Enrichment analysis was then conducted using the clusterProfiler package (v4.14.4), with a significance threshold of *p* < 0.05. To visualize the chromosomal distribution of the biomarkers, a chromosome localization map was generated using the RCircos package. Chromosomal position information for each gene was retrieved from the Ensembl database.^4^ To determine the subcellular localization of the biomarkers, their FASTA sequences were obtained from the UniProt database (see Footnote 1) and submitted to the WoLF PSORT server^5^ for prediction; the localization with the highest prediction score was adopted as the definitive subcellular assignment.

### 2.10 Immune infiltration analysis

To explore the immune infiltration profiles associated with the identified biomarkers, the relative abundances of various immune cell types in each sample from the GSE210666 dataset were estimated using the mmcp-counter algorithm implemented in the immunedeconv R package. Differences in immune cell infiltration between the BCAS and control groups were compared using the Wilcoxon rank-sum test. Furthermore, Spearman correlation analysis was performed to evaluate the associations between the biomarkers and the differentially infiltrated immune cells, with |ρ| > 0.3 and *p* < 0.05 considered statistically significant.

### 2.11 Construction of putative regulatory networks

To systematically elucidate the interactions among the identified biomarkers and their upstream regulatory mechanisms, a multi-layered regulatory network was constructed. First, a gene–gene interaction network was built using the GeneMANIA database to reveal functional associations among the biomarkers (19). Subsequently, the multiMiR R package (parameters: table = “tarbase,” org = “mmu”) was employed to integrate TarBase (v8.0) data for predicting miRNAs targeting these biomarkers (20). Furthermore, lncRNAs capable of interacting with the identified miRNAs were retrieved through the miRNet platform, thereby establishing a putative competing endogenous RNA (ceRNA) regulatory network comprising lncRNA–miRNA–mRNA axes. Concurrently, the RegNetwork module embedded within miRNet was utilized to systematically identify transcription factors (TFs) regulating the biomarkers, enabling the construction of TF–mRNA regulatory relationships (21). Finally, the putative TF–mRNA and ceRNA networks were integrated and visualized using Cytoscape.

### 2.12 Drug prediction and molecular docking

To systematically screen for potential therapeutic compounds against chronic cerebral hypoperfusion (CCH), the Comparative Toxicogenomics Database (CTD) was first queried to predict small-molecule chemicals interacting with the identified biomarkers, and compounds capable of targeting all candidate biomarkers simultaneously were selected for subsequent molecular docking validation (22). Three-dimensional protein structures were retrieved from the UniProt database (see Footnote 1), and ligand structures were obtained from PubChem.^6^ Receptor proteins were preprocessed using AutoDock tools, which involved the removal of water molecules and non-essential ligands and the addition of polar hydrogen atoms, followed by conversion to PDBQT format. Ligand molecules were similarly prepared by adding hydrogen atoms to define their flexible conformations. Molecular docking simulations were then performed using the AutoDock Vina algorithm (23). Docking poses were ranked by binding affinity, and the optimal conformation for each drug–target pair was selected. Finally, key interaction residues and binding modes between the compounds and their protein targets were visualized using PyMOL and LigPlot+ to elucidate the molecular mechanisms of action of the candidate compounds (24).

### 2.13 Statistical analysis

Quantitative data are presented as the mean ± standard deviation (SD). Behavioral data were statistically analyzed using GraphPad Prism (v10.0), and bioinformatics analyses were performed in R (v4.2). Between-group comparisons were conducted using the Mann–Whitney U test, with *p* < 0.05 considered statistically significant. For the immunofluorescence data, two cortical fields per animal were averaged to obtain one value per animal (*n* = 3 per group), and the results are presented descriptively because the sample size does not support nonparametric hypothesis testing.

## 3 Results

### 3.1 BCAS model mice displayed anxiety-like behavior and spatial memory deficits in behavioral tests

Figure 1 illustrates the overall data screening strategy and animal experiments. In the open field test (Figure 2L), the total distance traveled by BCAS mice (1,606 cm, *n* = 6) was significantly reduced compared with that of sham mice (2,462 cm, *n* = 6, *p* < 0.01), and the mean velocity was likewise significantly decreased (2.677 cm/s vs. 4.103 cm/s, *p* < 0.01), indicating diminished spontaneous locomotor and exploratory activity in BCAS mice (Figures 2H,I). BCAS mice exhibited a declining trend in both the frequency of entries into and cumulative duration spent in the central zone compared with sham mice, suggesting a tendency toward increased anxiety-like behavior; however, these differences did not reach statistical significance (*p* > 0.05), which may be attributable to the limited sample size (Figures 2J,K). In the Morris water maze acquisition phase (Figure 2G), escape latency declined over training days in both groups, suggesting intact basic spatial learning capacity; however, the BCAS group exhibited consistently longer escape latencies across all time points, reflecting a slower rate of spatial learning (Figure 2A). The swimming speed of BCAS mice was comparable to or even exceeded that of sham mice throughout the training period (day 1: 17.4 ± 2.0 cm/s vs. 14.3 ± 2.0 cm/s; day 5: 10.8 ± 1.7 cm/s vs. 7.2 ± 0.9 cm/s), confirming that the basic motor function of BCAS mice remained unimpaired and effectively ruling out motor deficits as a confounding factor in spatial memory assessment (Figures 2B–D). In the probe trial on day 6, neither swimming speed (18.54 ± 4.95 cm/s vs. 18.66 ± 1.59 cm/s, *p* > 0.05) nor total distance traveled (1,093.6 ± 282.3 cm vs. 1,119.9 ± 98.8 cm, *p* > 0.05) differed between the two groups, further corroborating the specificity of the spatial memory deficits. Regarding the core spatial memory indices, BCAS mice exhibited significantly fewer platform crossings (*p* < 0.01) and a prolonged latency to first target crossing (*p* < 0.05) (Figures 2E,F). Collectively, these findings demonstrate that BCAS mice present with reduced exploratory activity and spatial memory impairment that is not attributable to motor dysfunction, consistent with the typical behavioral phenotype of chronic cerebral hypoperfusion and the characteristic features of hypoperfusion-induced cognitive deficits (11).

**Figure 1:**
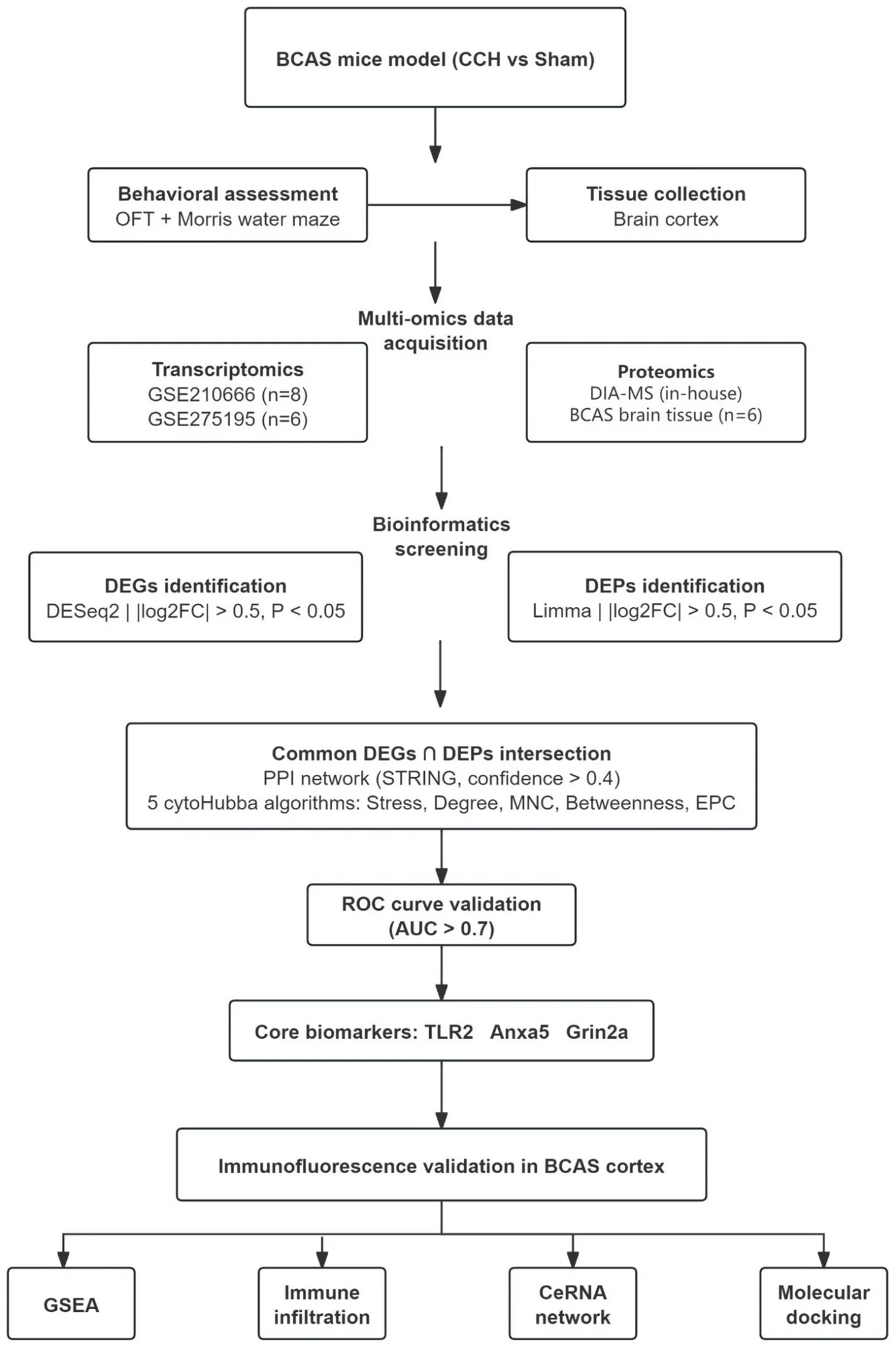
Workflow of the study, illustrating the multi-omics screening strategy, behavioral assessment, and immunofluorescence validation in the BCAS mouse model.

**Figure 2:**
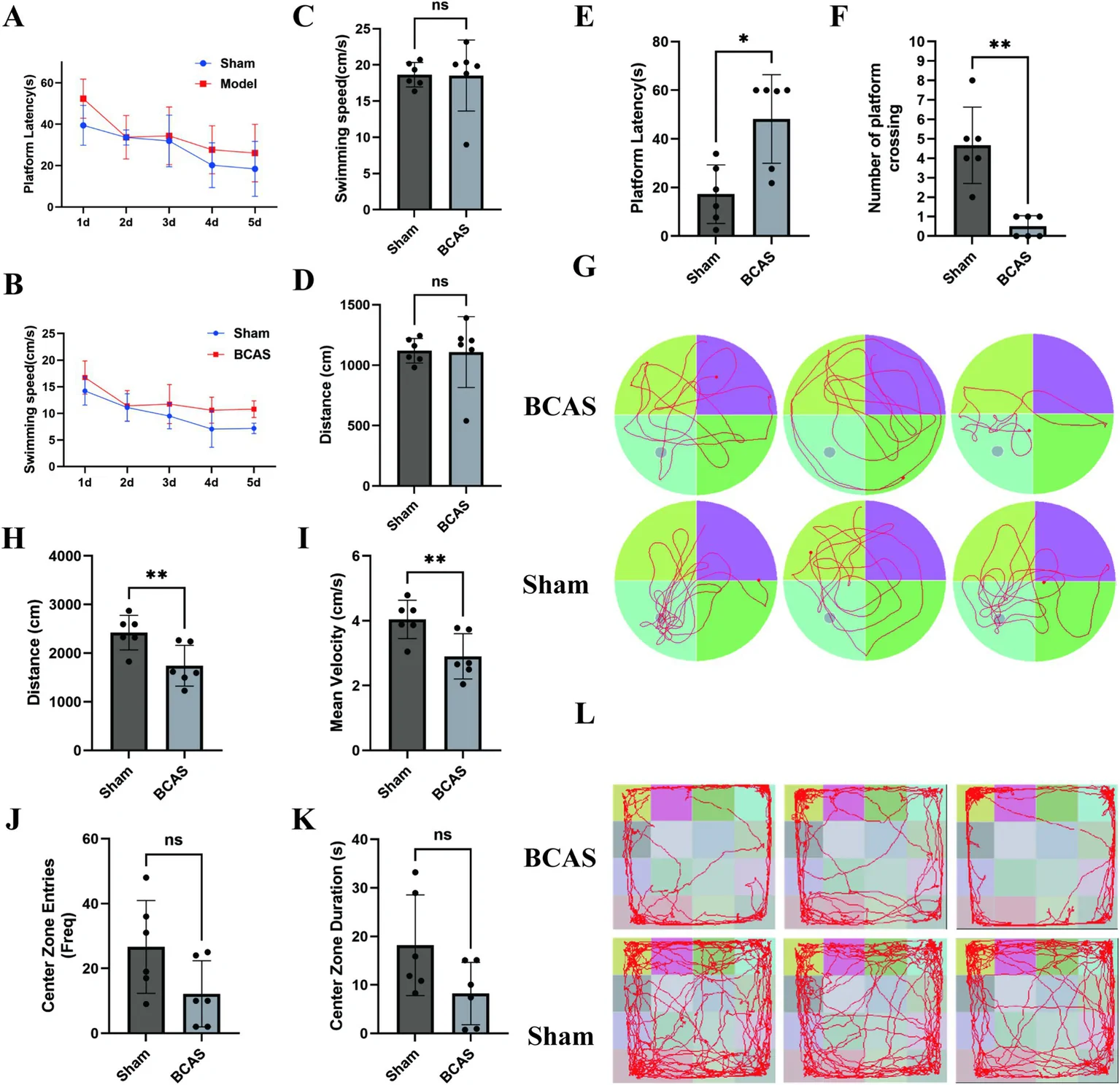
Behavioral assessment of BCAS mice. **(A)** MWM escape latency during acquisition phase. **(B)** Swimming velocity across training days. **(C,D)** Average speed and total distance in MWM training (ns). **(E,F)** Probe trial: latency to target crossing (*p < 0.05) and platform crossings (**p < 0.01); three BCAS mice failed to reach the target zone. **(G)** Representative probe trial trajectories of BCAS mice. **(H,I)** Total distance and mean velocity in OFT (**p < 0.01). **(J,K)** Center zone entry frequency and duration in OFT (ns). **(L)** Representative OFT trajectories (upper: BCAS; lower: Sham). *n* = 6 per group. Data are mean ± SD, analyzed by Mann–Whitney U test. *p < 0.05, **p < 0.01 vs. Sham; ns, not significant.

### 3.2 Identification and functional enrichment analysis of CCH-associated common DEGs

To identify DEGs associated with CCH, correlation analysis was first performed on samples from the GSE210666 and GSE275195 datasets. The results demonstrated a significant positive correlation between samples from the two datasets. PCA further showed a clear separation between disease and control samples in both datasets (Supplementary Figure S1). Subsequently, differential expression analysis revealed a total of 4,416 DEGs in the GSE210666 dataset, including 2,525 upregulated and 1,891 downregulated genes (Figures 3A,B). In the GSE275195 dataset, 3,478 DEGs were identified, comprising 2,442 upregulated and 1,036 downregulated genes (Figures 3C,D). Venn analysis was then performed to identify DEGs exhibiting consistent expression trends in both datasets. This analysis yielded 1,483 commonly upregulated genes (Figure 3E) and 393 commonly downregulated genes (Figure 3F).

**Figure 3:**
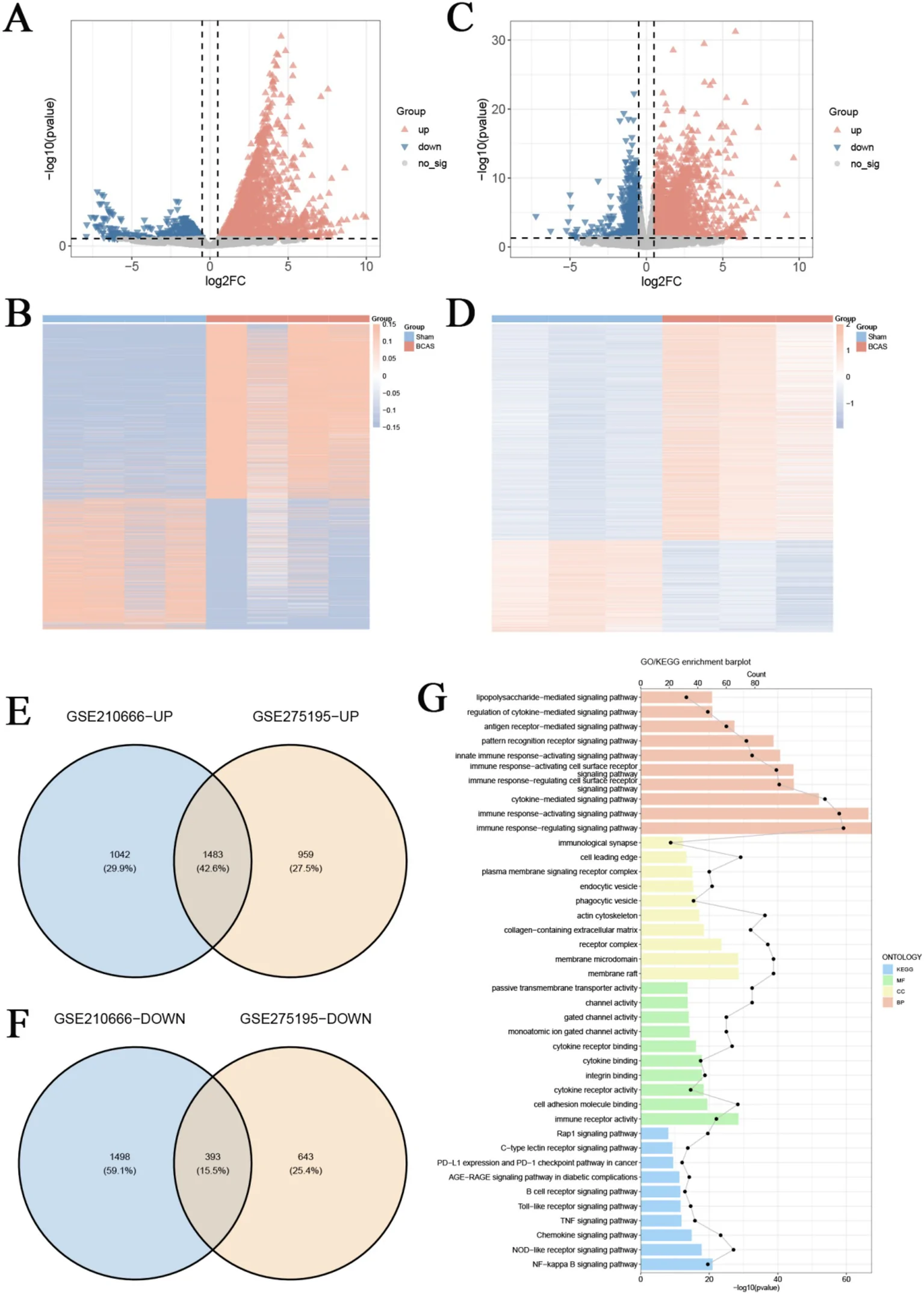
Identification of common differentially expressed genes (DEGs) in CCH transcriptomic datasets. **(A,B)** Volcano plot **(A)** and heatmap **(B)** showing DEGs in the GSE210666 dataset between CCH and control groups. **(C,D)** Volcano plot **(C)** and heatmap **(D)** showing DEGs in the GSE275195 dataset. **(E,F)** Venn diagrams illustrating the overlap of upregulated **(E)** and downregulated **(F)** DEGs between the two datasets. **(G)** GO and KEGG pathway enrichment analysis of the common DEGs.

To elucidate the biological functions of these common DEGs, functional enrichment analyses were conducted. The results showed that the genes were significantly enriched in 3,433 biological process (BP), 321 cellular component (CC), and 533 molecular function (MF) terms, as well as 152 KEGG pathways. Multiple immune- and inflammation-related pathways were significantly enriched, including regulation of cytokine-mediated signaling pathways, immune response–activating signaling pathways, the NF-κB signaling pathway, NOD-like receptor signaling pathway, chemokine signaling pathway, TNF signaling pathway, and Toll-like receptor signaling pathway (Figure 3G). These findings suggest that the common DEGs may contribute to the pathogenesis of CCH by modulating immune and inflammatory signaling pathways.

### 3.3 Proteomic profiling and functional enrichment analysis in CCH

To investigate proteomic alterations associated with CCH, inter-sample correlation analysis was first performed on the proteomic dataset, revealing a strong positive correlation among samples. PCA further demonstrated a clear separation between the disease and control groups (Supplementary Figure S2). Based on the predefined screening criteria, a total of 1,433 DEPs were identified, including 824 upregulated and 609 downregulated proteins (Figures 4A,B). To elucidate the biological functions of these DEPs, functional enrichment analyses were conducted. The results showed that the DEPs were significantly enriched in 2,623 BP, 354 CC, and 630 MF terms, as well as 105 KEGG pathways. Multiple pathways related to neuronal function and signal transduction were significantly enriched, including regulation of neuronal synaptic plasticity, regulation of neurotransmitter transport, GABAergic synapse, the cAMP signaling pathway, cGMP–PKG signaling pathway, GnRH signaling pathway, Rap1 signaling pathway, Ras signaling pathway, and pathways of neurodegeneration (Figure 4C).

**Figure 4:**
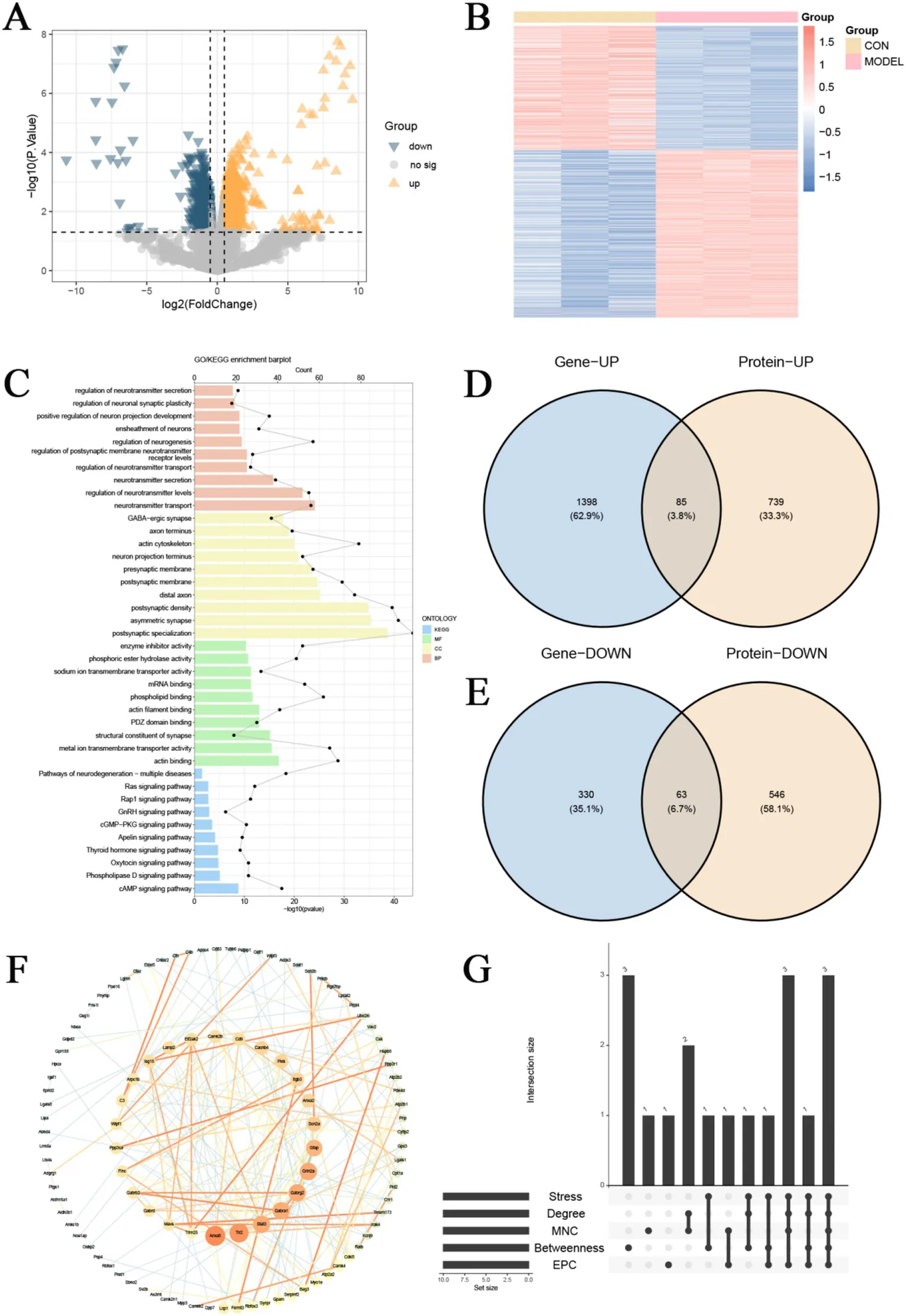
Proteomic profiling and identification of candidate genes in CCH. **(A,B)** Volcano plot **(A)** and heatmap **(B)** showing differentially expressed proteins (DEPs) between CCH and control groups. **(C)** KEGG pathway enrichment analysis of DEPs. **(D,E)** Venn diagrams showing the overlap of upregulated **(D)** and downregulated **(E)** DEGs and DEPs. **(F)** The PPI network for candidate genes. **(G)** Venn diagram illustrating the intersection of top-ranked genes identified by five cytoHubba algorithms (Stress, Degree, MNC, Betweenness, and EPC).

### 3.4 *Tlr2*, *Anxa5*, and *Grin2a* were identified as candidate biomarkers

To further identify potential biomarkers, the upregulated and downregulated DEGs/DEPs from the transcriptomic and proteomic datasets were intersected. This analysis yielded 85 commonly upregulated genes/proteins and 63 commonly downregulated genes/proteins (Figures 4D,E). The PPI network constructed for these candidate genes is shown in Figure 4F, comprising 107 nodes and 195 edges. To further screen for key biomarkers, five network topology algorithms were applied to rank the candidate genes (Table 1). The intersection of the top-ranked genes identified by these five algorithms was defined as the core biomarker set. Ultimately, three candidate biomarkers were identified: *Tlr2*, *Anxa5*, and *Grin2a* (Figure 4G). To validate the expression patterns and diagnostic performance of these three genes, their expression levels were evaluated in the two transcriptomic datasets (GSE210666 and GSE275195) as well as in the proteomic dataset using the Wilcoxon rank-sum test. Boxplot analyses showed that, compared with the control group, *Tlr2* and *Anxa5* were significantly upregulated in GSE210666 (Figure 5A), GSE275195 (Figure 5B), and the proteomic dataset (Figure 5C) (*p* < 0.05), whereas *Grin2a* was significantly downregulated (*p* < 0.05). Importantly, the expression trends of these three genes were consistent across all datasets. ROC curve analyses were subsequently performed to assess the diagnostic accuracy of each gene. The results demonstrated that in GSE210666 (Figure 5D), GSE275195 (Figure 5E), and the proteomic dataset (Figure 5F), *Tlr2*, *Anxa5*, and *Grin2a* all achieved AUC values greater than 0.7 in distinguishing the disease group from the control group, indicating good discriminative performance within these discovery datasets. Collectively, these findings suggest that *Tlr2*, *Anxa5*, and *Grin2a* may serve as candidate biomarkers for CCH.

**Table 1:** Genes obtained using different methods.

Stress	Degree	MNC	Betweenness	EPC
Gfap	Anxa5	Anxa5	Gfap	Anxa5
*TLR2*	*TLR2*	Gabrg2	*TLR2*	*TLR2*
Anxa5	*Grin2a*	Gabra1	Anxa5	Gfap
Plek	Gabrg2	*TLR2*	Plek	Stat3
*Grin2a*	Gabra1	*Grin2a*	*Grin2a*	Cd9
Itgb3	Stat3	Stat3	Ppp3ca	*Grin2a*
Gabrg2	Gfap	Isg15	Pld2	Gabrg2
Gabra1	Scn2a	Scn2a	C3	Gabra1
Anxa2	Cacnb4	Cacnb4	Itgb3	Anxa2
Stat3	Plek	Eif2ak2	Anxa2	Eif2ak2

**Figure 5:**
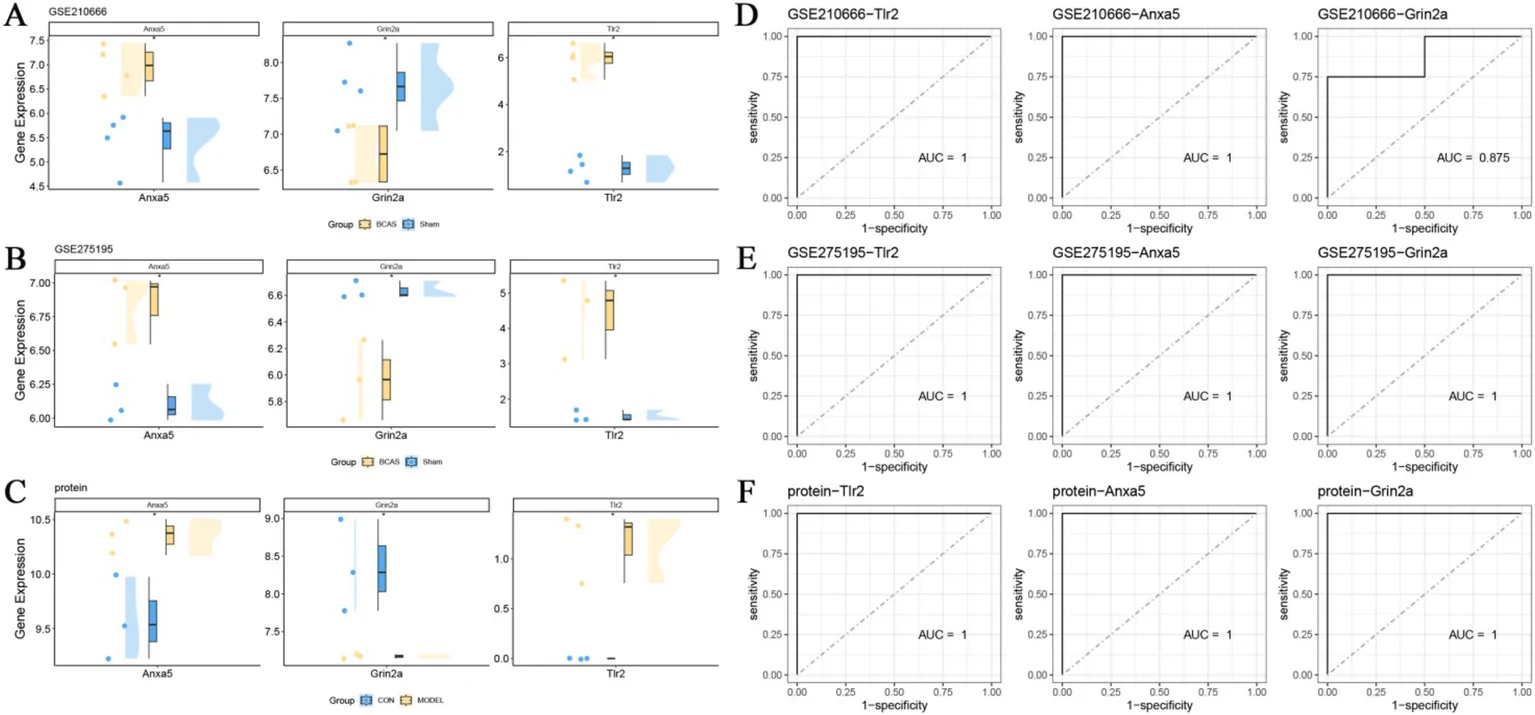
Expression analysis and discriminative performance of *Tlr2*, *Anxa5*, and *Grin2a* in CCH. **(A–C)** Boxplots showing the expression levels of *Tlr2*, *Anxa5*, and *Grin2a* in GSE210666 **(A)**, GSE275195 **(B)**, and the proteomic dataset **(C)**. Data are presented as box plots with individual data points (GSE210666: *n* = 4 per group; GSE275195 and the proteomic dataset: *n* = 3 per group). *p < 0.05, **p < 0.01, ***p < 0.001 by Wilcoxon rank-sum test. **(D–F)** Receiver operating characteristic (ROC) curves and corresponding area under the curve (AUC) values for *Tlr2*, *Anxa5*, and *Grin2a* in GSE210666 **(D)**, GSE275195 **(E)**, and the proteomic dataset **(F)**.

### 3.5 Immunofluorescence results

To validate the expression alterations of the candidate biomarkers at the protein and tissue levels, serial immunofluorescence staining was performed on brain tissue sections from BCAS model and sham-operated mice. Dual immunofluorescence staining for TLR2 and IBA1 revealed that the TLR2-positive area (red fluorescence) in the cerebral cortex was on average higher in the BCAS group than in the sham group (mean 6.27% ± 1.07% vs. 2.73% ± 0.67%; *n* = 3 per group) (Figures 6E,F), indicating upregulation of TLR2 protein under chronic hypoperfusion. Similarly, the IBA1-positive area was on average higher in the BCAS group (mean 4.94% ± 1.25% vs. 3.63% ± 0.45%) (Figure 6G), where positive cells exhibited a morphological shift from resting ramified to activated amoeboid morphology, indicative of microglial activation. Further co-localization analysis demonstrated prominent overlap of TLR2 and IBA1 fluorescent signals in the BCAS group, with the percentage of co-expressed positive area higher than that in the sham group (mean 3.00% ± 0.81% vs. 1.59% ± 0.54%) (Figure 6H), suggesting that TLR2 is predominantly expressed in activated microglia and infiltrating macrophages. This finding is consistent with the bioinformatic predictions regarding TLR2 function and provides histological evidence supporting TLR2 as a potential mediator of neuroinflammation in CCH.

**Figure 6:**
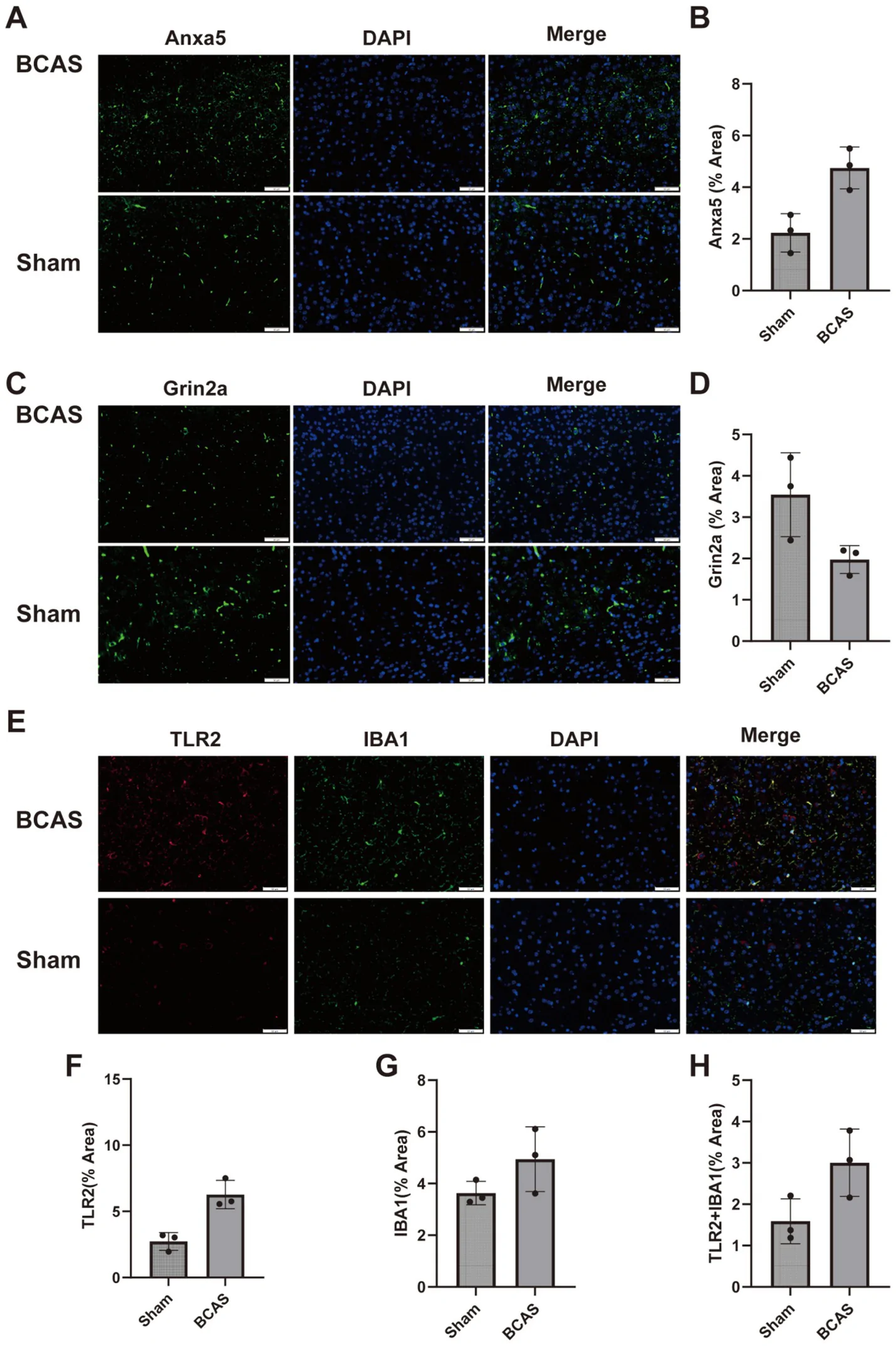
Immunofluorescence validation of the candidate biomarkers in BCAS cortex. **(A-B)** ANXA5 staining (green) and quantification, on average higher in BCAS. (C-D) GRIN2A staining (green) and quantification, on average lower in BCAS. **(E)** Dual TLR2/IBA1 immunofluorescence with DAPI. **(F-H)** Quantification of TLR2-positive area **(F)**, IBA1-positive area **(G)**, and TLR2/IBA1 co-localized area **(H)**, all on average higher in BCAS. TLR2-positive cells showed ramified-to-amoeboid morphological transition in BCAS mice. Scale bar = 50 μm. *n* = 3 per group; each symbol represents one animal (mean of two cortical fields). Because *n* = 3 per group does not support nonparametric hypothesis testing, the results are presented descriptively.

Additionally, the expression levels of ANXA5 and GRIN2A in the mouse cerebral cortex were examined. The percentage of ANXA5-positive area was on average higher in the BCAS group than in the sham group (mean 4.75% ± 0.81% vs. 2.24% ± 0.74%; *n* = 3 per group) (Figures 6A,B), whereas the percentage of GRIN2A-positive area, a key NMDA receptor subunit, was on average lower in the BCAS group (mean 1.97% ± 0.34% vs. 3.54% ± 1.01%) (Figures 6C,D). These results demonstrate that chronic cerebral ischemia induces cortical upregulation of ANXA5 and downregulation of GRIN2A at the tissue level, corroborating the findings from the multi-omics analysis. Given the limited sample size (*n* = 3 per group), these results are presented descriptively without formal statistical testing.

### 3.6 GSEA enrichment and cellular localization of *Tlr2*, *Anxa5*, and *Grin2a*

GSEA revealed that *Tlr2*, *Anxa5*, and *Grin2a* were commonly enriched in several immune- and inflammation-related KEGG pathways, including the Toll-like receptor signaling pathway, JAK–STAT signaling pathway, chemokine signaling pathway, B cell receptor signaling pathway, NOD-like receptor signaling pathway, p53 signaling pathway, T cell receptor signaling pathway, Notch signaling pathway, and RIG-I-like receptor signaling pathway (Figure 7A). In addition, GSEA of *Grin2a* demonstrated significant enrichment in multiple GOBP terms associated with neuronal and immune functions, such as neurotransmitter secretion, cytokine-mediated signaling pathway, immune response–regulating signaling pathway, Toll-like receptor signaling pathway, neurotransmitter transport, regulation of neurotransmitter levels, antigen receptor–mediated signaling pathway, and regulation of neurotransmitter receptor activity (Figure 7B).

**Figure 7:**
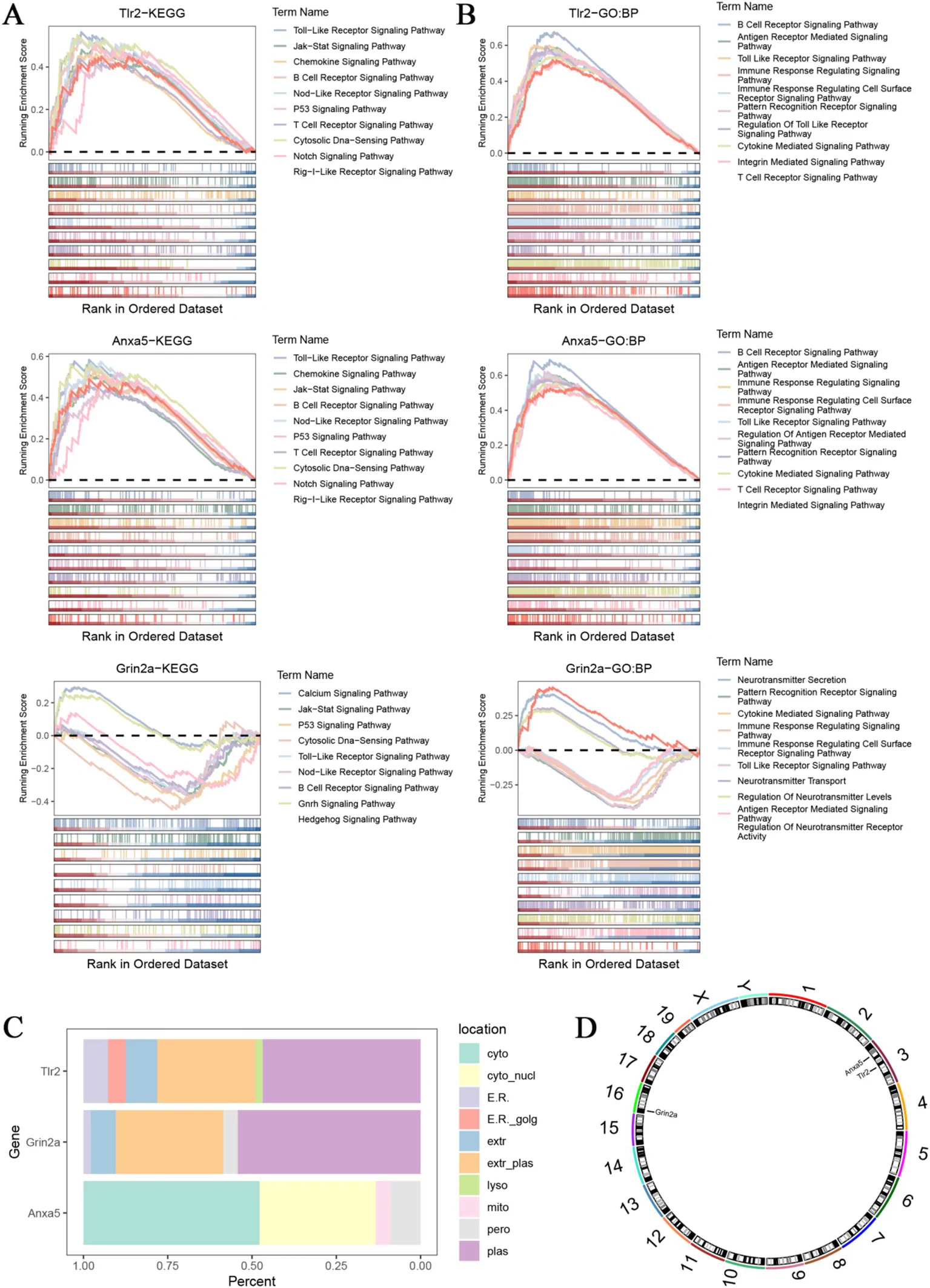
Functional enrichment and subcellular localization of *Tlr2*, *Anxa5*, and *Grin2a*. **(A)** GSEA showing common enrichment of *Tlr2*, *Anxa5*, and *Grin2a* in immune- and inflammation-related KEGG pathways. **(B)** GSEA of *Grin2a* revealing enrichment in GOBP terms associated with neuronal and immune functions. **(C)** Subcellular localization prediction of TLR2, ANXA5, and GRIN2A. **(D)** Chromosomal distribution of *Tlr2*, *Anxa5*, and *Grin2a*.

Subcellular localization analysis indicated that TLR2 was predominantly localized to the plasma membrane, ANXA5 was mainly distributed in the cytoplasm, and GRIN2A was primarily localized to the plasma membrane (Figure 7C). The chromosomal locations of *Tlr2*, *Anxa5*, and *Grin2a* were shown in Figure 7D.

### 3.7 Immune cell association with biomarkers

Given the crucial role of immune responses in disease progression, immune cell infiltration in control and disease samples was evaluated. The heatmap illustrates the relative abundance and distribution of immune cell populations across individual samples (Figure 8A). Subsequent comparative analysis revealed significant differences in five immune cell subtypes between the control and disease groups. Specifically, basophils, macrophages/monocytes, natural killer (NK) cells, total T cells, and CD8^+^ T cells showed significantly altered infiltration levels in disease samples compared with controls (*p* < 0.05; Figure 8B). Furthermore, correlation analysis was performed to investigate the association between the expression of the three identified biomarkers and the infiltration levels of these differentially abundant immune cell subsets. As shown in Figure 8C, *Tlr2* and *Anxa5* expression levels were significantly positively correlated with the infiltration of basophils, macrophages/monocytes, NK cells, total T cells, and CD8^+^ T cells (*p* < 0.05). In contrast, *Grin2a* expression was significantly negatively correlated with the infiltration of these immune cell populations.

**Figure 8:**
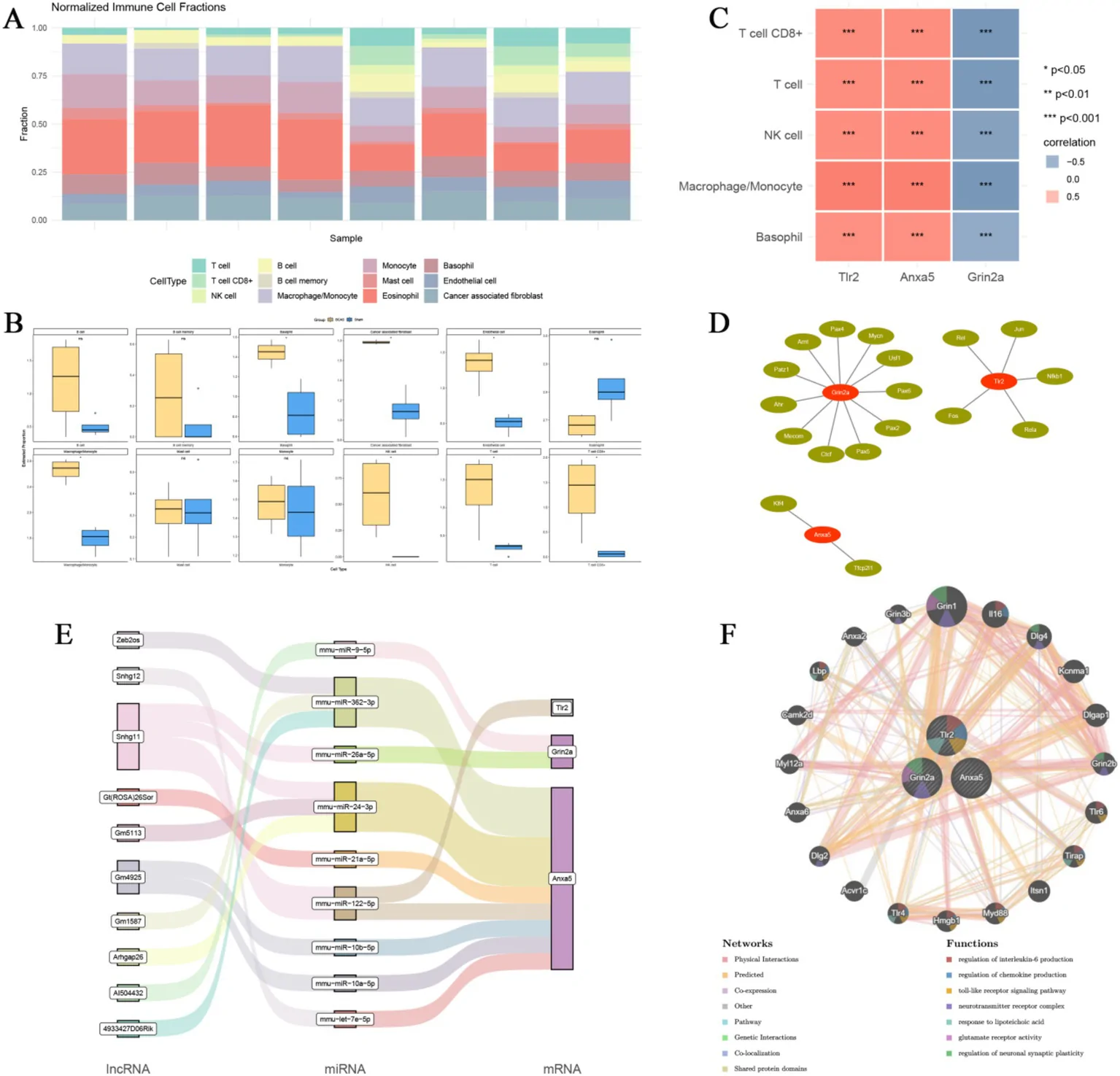
Immune infiltration and regulatory network analysis of biomarkers in CCH. **(A)** Heatmap showing the relative abundance of immune cell subsets in individual samples from GSE210666. **(B)** Comparison of immune cell infiltration levels between CCH and control groups. *p < 0.05 by Wilcoxon rank-sum test. **(C)** Spearman correlation analysis between biomarker expression and differential immune cell infiltration. Red indicates positive correlation, blue indicates negative correlation. *p < 0.05, **p < 0.01, ***p < 0.001. **(D)** Transcription factor-mRNA regulatory network of *Tlr2*, *Anxa5*, and *Grin2a*. Triangles represent transcription factors, circles represent biomarkers. **(E)** Competing endogenous RNA (ceRNA) network comprising biomarkers, miRNAs, and lncRNAs. **(F)** GeneMANIA network showing interactions between the three biomarkers and 20 highly associated genes, with functional enrichment analysis results.

### 3.8 Regulatory network analysis of the candidate biomarkers and associated pathways

The mRNA–TF regulatory network consisted of 21 nodes, including three biomarkers and 18 TFs, connected by 18 regulatory interactions (Figure 8D). In addition, a putative ceRNA regulatory network was constructed comprising 22 nodes (three biomarkers, nine miRNAs, and 10 lncRNAs) and 25 interaction edges. Topological analysis of the putative ceRNA network revealed that *Anxa5* exhibited the highest degree among mRNAs, while mmu-miR-24-3p and Snhg11 showed the highest degree values among miRNAs and lncRNAs, respectively. Reciprocal regulatory relationships were observed among these key nodes (Figure 8E), suggesting that these predicted regulatory axes may be involved in disease progression and require experimental validation. Furthermore, GeneMANIA analysis demonstrated extensive interactions between the three hub genes and 20 highly associated genes. Functional enrichment analysis indicated that these genes were predominantly involved in immune- and inflammation-related processes, including regulation of interleukin-6 production, regulation of chemokine production, and the Toll-like receptor signaling pathway. Enrichment was also observed in neural-related pathways such as neurotransmitter receptor complex, response to lipoteichoic acid, glutamate receptor activity, and regulation of neuronal synaptic plasticity (Figure 8F).

### 3.9 Identification of potential therapeutic compounds and molecular docking validation

To explore potential therapeutic agents associated with the disease, the CTD was utilized to predict drugs related to the identified biomarkers. Based on these biomarkers, a biomarker–drug interaction network was constructed and visualized (Figure 9A). Subsequently, three compounds shared by all three genes—pirinixic acid, particulate matter, and bisphenol A—were selected for molecular docking analysis (Figure 9B). Among these compounds, particulate matter was excluded from docking analysis due to the absence of an available three-dimensional chemical structure in the PubChem database. The molecular docking results were summarized in Table 2. Favorable binding interactions were observed between TLR2 and pirinixic acid (binding energy: −7.3 kcal/mol), as well as between ANXA5 and pirinixic acid (binding energy: −7.5 kcal/mol). In addition, bisphenol A (BPA) exhibits strong binding affinity with GRIN2A (−7.7 kcal/mol).Figure 9C illustrated the optimal docking conformations with the lowest binding energies for each gene–compound pair and highlights the key amino acid residues at the binding sites.

**Figure 9:**
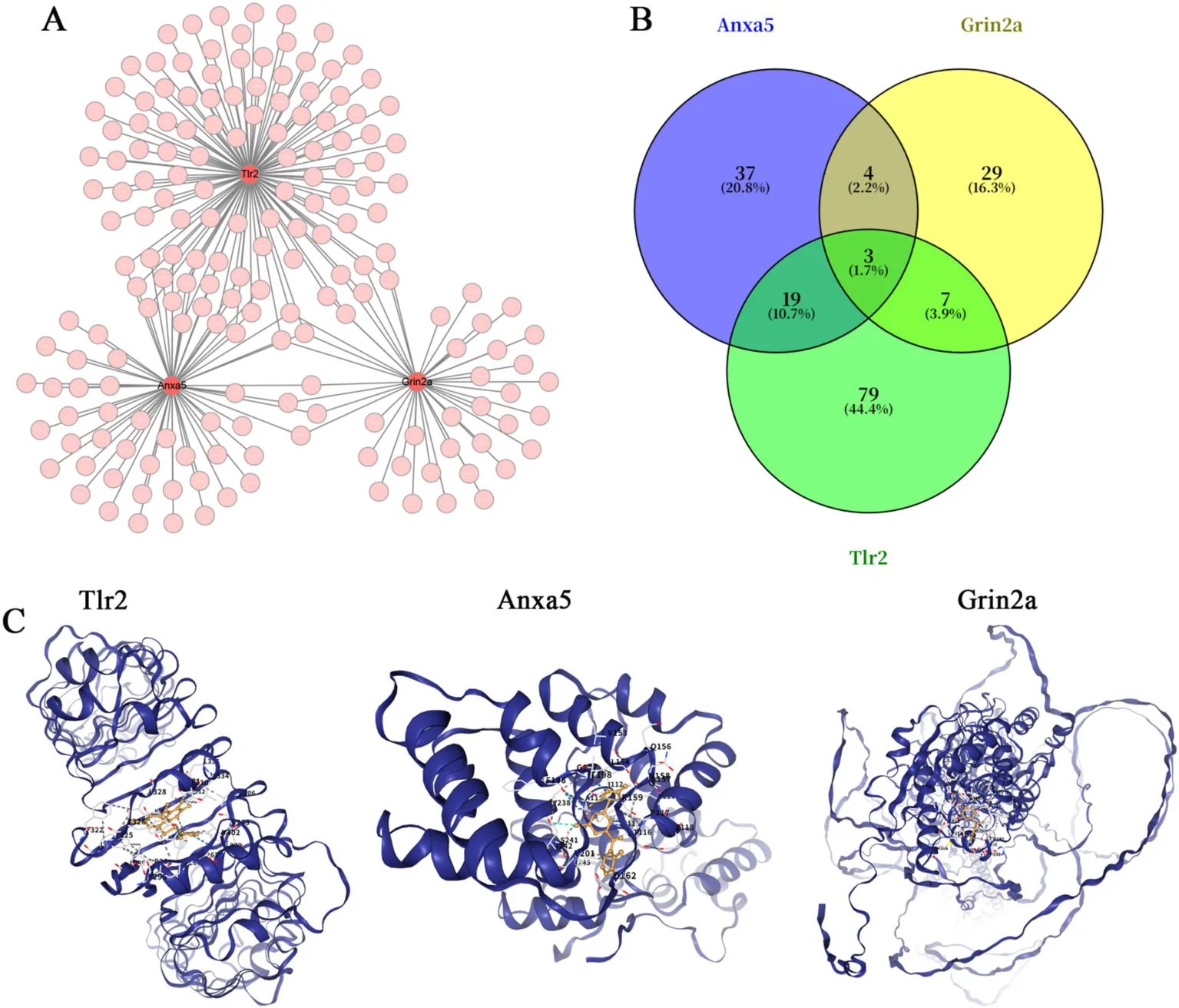
Drug prediction and molecular docking validation. **(A)** Biomarker-drug interaction network constructed using the CTD database. **(B)** Venn diagram showing three compounds shared by *Tlr2*, *Anxa5*, and *Grin2a*: pirinixic acid, particulate matter, and bisphenol A. **(C)** Molecular docking models showing the optimal binding conformations of pirinixic acid with TLR2 (left) and ANXA5 (middle), and bisphenol A with GRIN2A (right). Key amino acid residues involved in hydrogen bonding and hydrophobic interactions are highlighted.

**Table 2:** Molecular docking binding energy.

Gene	Drug	Binding energy (kcal/mol)
TLR2	Pirinixic acid	−7.3
TLR2	Bisphenol A	−7.2
Anxa5	Pirinixic acid	−7.5
Anxa5	Bisphenol A	−7.3
Grin2a	Pirinixic acid	−7.4
Grin2a	Bisphenol A	−7.7

## 4 Discussion

Chronic cerebral hypoperfusion (CCH) is a complex disorder driven by multifactorial and multi-layered pathogenic interactions. In the present study, we integrated transcriptomic (RNA-seq) and proteomic (DIA-MS) data, combined with protein–protein interaction (PPI) network analysis and intersecting screening using five topological algorithms (Stress, Degree, MNC, Betweenness, and EPC), to systematically identify candidate biomarkers occupying critical hub positions within the network—*Tlr2*, *Anxa5*, and *Grin2a*. We further evaluated the concordance of their expression trends across transcriptomic and proteomic levels, as well as their discriminative performance (AUC > 0.7) within these discovery datasets, and assessed their expression alterations at the tissue level via immunofluorescence staining. Immune infiltration profiling revealed significant associations between these biomarkers and key immune cell subsets. Building upon these findings, upstream regulatory network construction and molecular docking analyses provided preliminary insights into the regulatory mechanisms and potential targeting compounds, thereby offering novel perspectives on the pathogenesis of CCH.

### 4.1 Potential molecular mechanisms and functional implications of core biomarkers *Tlr2*, *Anxa5*, and *Grin2a*

In the present study, *Tlr2* and *Anxa5* were significantly upregulated, whereas *Grin2a* was significantly downregulated, in the BCAS mouse model, with concordant expression trends observed across the two transcriptomic datasets and the proteomic dataset, underscoring the consistency of these alterations. ROC analysis further revealed that all three genes achieved AUC values > 0.7, indicating favorable discriminative ability within these discovery datasets. TLR2, a key pattern recognition receptor of the innate immune system, plays a pivotal role in recognizing diverse endogenous danger-associated molecular patterns (DAMPs), including HMGB1 and heat shock proteins released from damaged or necrotic cells (25–27). In the context of cerebral ischemia, the massive release of DAMPs can sustain activation of the TLR2–MyD88–NF-κB signaling axis, triggering cascaded secretion of pro-inflammatory cytokines (TNF-α, IL-1β, and IL-6) and thereby exacerbating neuroinflammatory responses (28, 29). Annexin A5 (ANXA5) is a calcium-dependent phospholipid-binding protein with high affinity for phosphatidylserine (PS) (30). The role of ANXA5 in cerebral ischemia exhibits a multifaceted duality: on the one hand, ANXA5 may participate in intracellular calcium overload and annexin crystal lattice formation on damaged membranes during the early phase; on the other hand, its PS-binding property can be harnessed to modulate inflammatory phagocytosis at a later stage (31, 32).

GRIN2A (GluN2A) is a critical functional subunit of NMDA-type glutamate receptors (NMDARs), playing a central role in regulating synaptic transmission strength and modulating synaptic plasticity processes such as long-term potentiation (LTP) and long-term depression (LTD) (33). The high calcium permeability of NMDARs renders them key upstream regulators of learning- and memory-associated signaling cascades, including the CaMKII–CREB–BDNF axis (34). In the context of cerebral ischemia, glutamate excitotoxicity represents the primary mechanism of acute neuronal death; however, under chronic hypoperfusion, persistent alterations in synaptic transmission likewise profoundly compromise cognitive function (35, 36). The differential expression pattern of *Grin2a* observed in the present study, in conjunction with the significant spatial memory impairment demonstrated by BCAS mice in the Morris water maze and the reduced spontaneous exploratory activity in the open field test, suggests that GRIN2A-mediated impairment of synaptic plasticity may constitute an important molecular substrate underlying CCH-induced cognitive dysfunction. Collectively, these three biomarkers delineate the pathogenic mechanisms of CCH from three distinct yet interrelated dimensions—neuroinflammation, cellular stress, and synaptic function—and their combined detection may serve as a candidate biomarker panel for CCH diagnosis, pending independent validation.

### 4.2 Immune infiltration and neurovascular unit destabilization

Immune infiltration profiling revealed markedly altered immune cell infiltration levels in the BCAS mouse model, encompassing not only significant changes in brain-resident innate immune cells (microglia/macrophages) but also a notable increase in cerebral infiltration of peripheral adaptive immune cells (CD8^+^ T cells). This finding aligns with the growing recognition of the role of the neurovascular unit (NVU) in cerebral ischemia: BBB disruption induced by chronic hypoperfusion constitutes a prerequisite for peripheral immune cell infiltration, and the infiltrated immune cells, in turn, further exacerbate BBB damage and neuroinflammation, thereby establishing a vicious cycle (37, 38). Correlation analysis demonstrated that the expression levels of *Tlr2* and *Anxa5* were positively associated with the infiltration of the aforementioned immune cells, whereas *Grin2a* exhibited a negative correlation, suggesting that these three genes may participate in CCH-associated neuroinflammatory responses through modulating immune cell recruitment or activation. As a pattern recognition receptor, TLR2 can directly recognize damage-associated molecular patterns and activate downstream inflammatory pathways; ANXA5 may influence immune cell clearance through regulating phosphatidylserine exposure; and GRIN2A may indirectly modulate the immune microenvironment by affecting neuron–immune cell signaling crosstalk. These findings provide novel insights into the mechanisms underlying neuroimmune interactions in CCH. The co-localization of TLR2 *with* IBA1 in activated microglia, as demonstrated by our immunofluorescence findings, further corroborates the intimate link between TLR2 upregulation and neuroinflammatory activation within the CCH microenvironment.

### 4.3 Regulatory network analysis reveals multi-layered molecular regulatory mechanisms

The putative ceRNA regulatory network constructed in the present study suggests possible molecular mechanisms by which lncRNAs may indirectly modulate the expression of *Tlr2*, *Anxa5*, and *Grin2a* through competitive binding to miRNAs. The NEAT1/miR-21-5p/*Tlr2* and MALAT1/miR-146a-5p/*Anxa5* axes have been partially validated in various inflammation and ischemic injury models, suggesting that these regulatory axes may be mechanistically relevant in CCH (39, 40). GeneMANIA analysis revealed extensive interactions between the three core genes and 20 highly associated genes, with functional enrichment analysis indicating that these interacting genes are predominantly involved in immune-inflammatory and neurofunctional pathways. The identification of the ceRNA network provides candidate targets for future intervention studies, and offers a framework for understanding the molecular pathology of CCH. Moreover, the TF–mRNA regulatory network identified several transcription factors that may orchestrate the expression of the candidate biomarkers, further underscoring the multi-layered nature of transcriptional regulation in CCH. Integration of the ceRNA and TF–mRNA networks suggested that both epigenetic and transcriptional mechanisms may converge on the regulation of *Tlr2*, *Anxa5*, and *Grin2a*, highlighting the complexity of the regulatory architecture underlying CCH pathogenesis.

### 4.4 Analysis of the translational significance and limitations of drug prediction and molecular docking

Based on the CTD database, three compounds commonly targeting *Tlr2*, *Anxa5*, and *Grin2a* were predicted and screened. Molecular docking analyses revealed that pirinixic acid exhibited strong binding affinity with both TLR2 and ANXA5 (binding energies of −7.3 and −7.5 kcal/mol, respectively), suggesting its potential as a therapeutic candidate for CCH. Bisphenol A (BPA) demonstrated the strongest binding affinity with GRIN2A (−7.7 kcal/mol), implying that this ubiquitous endocrine disruptor—widely present in daily consumer products—may interfere with synaptic transmission through direct binding to the NMDA receptor subunit, thereby exacerbating the risk of cognitive impairment in patients with chronic cerebral hypoperfusion. This finding provides molecular-level evidence from a novel target perspective for environmental health research (41).

### 4.5 Limitations

Nevertheless, several limitations of the present study should be acknowledged. Firstly, the relatively small sample size may limit the statistical power of the analyses, and further validation with larger cohorts is warranted to confirm the multi-omics findings. Secondly, the molecular docking results are inherently computational predictions; the biological activity of the identified ligands requires further validation through *in vitro* and *in vivo* experiments. Furthermore, the GEO datasets utilized in this study were all derived from mouse models, and the translational relevance of these findings to human CCH remains to be established using clinical specimens. In addition, because GSE275195 and the in-house proteomic dataset participated in the initial candidate intersection step, the expression comparisons and ROC analyses performed in these datasets represent internal consistency and concordance checks rather than independent external validation; the diagnostic performance metrics should therefore be interpreted within this discovery framework, and formal validation will require an independent external cohort. Lastly, the predicted lncRNA–miRNA–mRNA regulatory interaction network is entirely based on database predictions and awaits experimental validation. Additionally, the BCAS model, while widely employed, may not fully recapitulate the multifactorial pathophysiology of human CCH. Moreover, cerebral blood flow was not directly measured in our BCAS cohort (e.g., by laser Doppler flowmetry or arterial spin labeling); therefore, the achieved degree of hypoperfusion was not quantified. Although the observed behavioral deficits and molecular alterations are consistent with published BCAS studies supporting model validity, they should not be interpreted as strictly dose-dependent effects of cerebral blood flow reduction, and future studies will incorporate direct blood flow monitoring.

## 5 Conclusion

Through the integration of transcriptomic and proteomic multi-omics data, construction of a PPI network, and cross-validation using five cytoHubba algorithms, the present study systematically identified three candidate biomarkers of chronic cerebral hypoperfusion, *Tlr2*, *Anxa5*, and *Grin2a*, all of which showed favorable discriminative performance (AUC > 0.70) within the discovery datasets. Behavioral assessments in the BCAS mouse model confirmed significant locomotor impairment and spatial cognitive deficits (Morris water maze). Immunofluorescence staining provided supportive histological evidence at the tissue level. GSEA, immune infiltration profiling, and putative ceRNA network construction further suggested the biological functional contexts of the candidate biomarkers, while in silico molecular docking analyses predicted that all three targets possess druggable binding capacity. Collectively, these findings suggest that *Tlr2*, *Anxa5*, and *Grin2a* represent candidate multi-omics signature molecules of CCH in the mouse brain, with consistent alterations at the transcriptomic, proteomic, and tissue levels. These hypothesis-generating findings provide a reference framework for future mechanistic studies and the development of diagnostic and therapeutic strategies, but require independent validation. However, these associations are not causal and require functional and human validation; *TLR2*, *ANXA5*, and *GRIN2A* correspond to neuroinflammation, barrier protection, and excitotoxicity, respectively, supporting future targeted validation and potential disease-modifying strategies in CCH.

## Data Availability

Transcriptomic datasets are available in GEO (GSE210666, GSE275195). The proteomic data can be requested from the authors upon request. All other data are available from the corresponding author upon reasonable request.

## Footnotes

1
http://www.uniprot.org

2
https://www.ncbi.nlm.nih.gov/geo/

3
https://cn.string-db.org/

4
http://asiadb.ensembl.org

5
https://wolfpsort.hgc.jp/

6
https://pubchem.ncbi.nlm.nih.gov/

## Supplementary material

SUPPLEMENTARY FIGURE S1 Correlation and principal component analysis of the transcriptomic datasets. **(A)** Sample-to-sample correlation heatmap and **(B)** principal component analysis plot showing clear separation between CCH and control samples in GSE210666 and GSE275195.

SUPPLEMENTARY FIGURE S2 Correlation and principal component analysis of the in-house DIA proteomic dataset. **(A)** Sample-to-sample correlation heatmap and **(B)** principal component analysis plot showing clear separation between model and control samples.

SUPPLEMENTARY Table S1 Raw per-animal immunofluorescence quantification values (percentage of positive tissue area for ANXA5, GRIN2A, TLR2, IBA1, and TLR2/IBA1 co-localization) in the sham and BCAS groups.

## Glossary

CCH: Chronic cerebral hypoperfusion
BCAS: Bilateral common carotid artery stenosis
TLR2: Toll-like receptor 2
ANXA5: Annexin A5
GRIN2A: Glutamate ionotropic receptor NMDA type subunit 2A
DIA: Data-independent acquisition
MS: Mass spectrometry
PPI: Protein–protein interaction
DEG: Differentially expressed gene
DEP: Differentially expressed protein
GO: Gene Ontology
KEGG: Kyoto Encyclopedia of Genes and Genomes
GSEA: Gene set enrichment analysis
ROC: Receiver operating characteristic curve
AUC: Area under the curve
ceRNA: Competing endogenous RNA
miRNA: MicroRNA
lncRNA: Long non-coding RNA
TF: Transcription factor
CTD: Comparative Toxicogenomics Database
BPA: Bisphenol A
MWM: Morris water maze
OFT: Open field test
IBA1: Ionized calcium-binding adapter molecule 1
