# Supplementary material for: Cross-dataset transcriptomics–proteomics analysis identifies Tlr2, Anxa5, and Grin2a as candidate mouse-brain signatures associated with chronic cerebral hypoperfusion
Source: Front Neurol. 2026 Oct 1;17:1911864. doi: 10.3389/fneur.2026.1911864 (PMC13668749; doi:10.3389/fneur.2026.1911864)
Supplement: Supplementary file 1 [file Figure_1.docx]

**Supplementary Figure 1. Sample correlation and principal component analysis of transcriptomic datasets.** (A) Sample correlation heatmap of GSE210666 and GSE275195 datasets. (B) Principal component analysis (PCA) plots showing the separation between CCH and control samples in GSE210666 (left) and GSE275195 (right).
