# Supplementary material for: Cross-dataset transcriptomics–proteomics analysis identifies Tlr2, Anxa5, and Grin2a as candidate mouse-brain signatures associated with chronic cerebral hypoperfusion
Source: Front Neurol. 2026 Oct 1;17:1911864. doi: 10.3389/fneur.2026.1911864 (PMC13668749; doi:10.3389/fneur.2026.1911864)
Supplement: Supplementary file 2 [file Figure_2.docx]

**Supplementary Figure 2. Sample correlation and principal component analysis of proteomic dataset.** (A) Sample correlation heatmap of the proteomic dataset. (B) PCA plot showing the separation between CCH and control samples in the proteomic dataset.
