# Supplementary material for: Cross-dataset transcriptomics–proteomics analysis identifies Tlr2, Anxa5, and Grin2a as candidate mouse-brain signatures associated with chronic cerebral hypoperfusion
Source: Front Neurol. 2026 Oct 1;17:1911864. doi: 10.3389/fneur.2026.1911864 (PMC13668749; doi:10.3389/fneur.2026.1911864)
Supplement: Supplementary file 3 [file Table_1.docx]

**Supplementary Table S1.** Per-animal immunofluorescence quantification in the mouse cerebral cortex.

Values are the percentage of positive tissue area (% area) in the cerebral cortex. For each animal, two non-overlapping 200× cortical fields were analysed with the Halo image analysis platform and the two measurements were averaged to give a single value per animal. The per-animal value was the unit of statistical analysis (biological replicates: sham, n = 3; BCAS, n = 3). Sham animals were AS611, AS621 and AS628; BCAS animals were AS605, AS617 and AS623. Because n = 3 per group does not support nonparametric hypothesis testing, the immunofluorescence results are presented descriptively and no P values are reported. Mean ± SD in the summary rows were computed across the three animals per group; the values reported in the manuscript are rounded to two decimal places.

| **Anxa5 (% positive area)** | | | | |
| --- | --- | --- | --- | --- |
| **Group** | **Animal ID** | **Field 1 (% area)** | **Field 2 (% area)** | **Mean per animal (% area)** |
| Sham | AS611 | 3.372460 | 2.485350 | 2.928905 |
| Sham | AS621 | 1.369229 | 1.530315 | 1.449772 |
| Sham | AS628 | 2.610860 | 2.046301 | 2.328581 |
| BCAS | AS605 | 4.018985 | 3.764834 | 3.891910 |
| BCAS | AS617 | 6.356341 | 4.637429 | 5.496885 |
| BCAS | AS623 | 6.311279 | 3.386975 | 4.849127 |
| **Sham** | **Mean ± SD** | 2.451 ± 1.011 | 2.021 ± 0.478 | **2.236 ± 0.744** |
| **BCAS** | **Mean ± SD** | 5.562 ± 1.337 | 3.930 ± 0.641 | **4.746 ± 0.807** |
| **Grin2a (% positive area)** | | | | |
| **Group** | **Animal ID** | **Field 1 (% area)** | **Field 2 (% area)** | **Mean per animal (% area)** |
| Sham | AS611 | 4.621776 | 4.259671 | 4.440724 |
| Sham | AS621 | 3.560963 | 3.931364 | 3.746164 |
| Sham | AS628 | 2.065094 | 2.818147 | 2.441621 |
| BCAS | AS605 | 2.151889 | 1.021048 | 1.586469 |
| BCAS | AS617 | 2.389913 | 1.882180 | 2.136047 |
| BCAS | AS623 | 1.377396 | 3.013357 | 2.195377 |
| **Sham** | **Mean ± SD** | 3.416 ± 1.284 | 3.670 ± 0.756 | **3.543 ± 1.015** |
| **BCAS** | **Mean ± SD** | 1.973 ± 0.529 | 1.972 ± 0.999 | **1.973 ± 0.336** |
| **TLR2 (% positive area)** | | | | |
| **Group** | **Animal ID** | **Field 1 (% area)** | **Field 2 (% area)** | **Mean per animal (% area)** |
| Sham | AS611 | 4.629650 | 1.750218 | 3.189934 |
| Sham | AS621 | 1.656394 | 2.266015 | 1.961205 |
| Sham | AS628 | 3.224371 | 2.846607 | 3.035489 |
| BCAS | AS605 | 5.357382 | 9.643894 | 7.500638 |
| BCAS | AS617 | 4.185668 | 6.929628 | 5.557648 |
| BCAS | AS623 | 4.848985 | 6.656978 | 5.752982 |
| **Sham** | **Mean ± SD** | 3.170 ± 1.487 | 2.288 ± 0.549 | **2.729 ± 0.669** |
| **BCAS** | **Mean ± SD** | 4.797 ± 0.588 | 7.744 ± 1.651 | **6.270 ± 1.070** |
| **IBA1 (% positive area)** | | | | |
| **Group** | **Animal ID** | **Field 1 (% area)** | **Field 2 (% area)** | **Mean per animal (% area)** |
| Sham | AS611 | 3.723851 | 2.858371 | 3.291111 |
| Sham | AS621 | 4.091938 | 2.809609 | 3.450774 |
| Sham | AS628 | 4.683345 | 3.609155 | 4.146250 |
| BCAS | AS605 | 4.231299 | 3.002475 | 3.616887 |
| BCAS | AS617 | 4.703552 | 5.505376 | 5.104464 |
| BCAS | AS623 | 5.011398 | 7.202089 | 6.106744 |
| **Sham** | **Mean ± SD** | 4.166 ± 0.484 | 3.092 ± 0.448 | **3.629 ± 0.455** |
| **BCAS** | **Mean ± SD** | 4.649 ± 0.393 | 5.237 ± 2.113 | **4.943 ± 1.253** |
| **TLR2/IBA1 co-localized (% positive area)** | | | | |
| **Group** | **Animal ID** | **Field 1 (% area)** | **Field 2 (% area)** | **Mean per animal (% area)** |
| Sham | AS611 | 1.842240 | 0.901340 | 1.371790 |
| Sham | AS621 | 1.188411 | 1.182244 | 1.185328 |
| Sham | AS628 | 2.648143 | 1.764638 | 2.206391 |
| BCAS | AS605 | 2.131208 | 2.187654 | 2.159431 |
| BCAS | AS617 | 2.563901 | 3.569596 | 3.066749 |
| BCAS | AS623 | 3.421696 | 4.144590 | 3.783143 |
| **Sham** | **Mean ± SD** | 1.893 ± 0.731 | 1.283 ± 0.440 | **1.588 ± 0.544** |
| **BCAS** | **Mean ± SD** | 2.706 ± 0.657 | 3.301 ± 1.006 | **3.003 ± 0.814** |

*SD, standard deviation; BCAS, bilateral common carotid artery stenosis.*
